# Eosinophilic granulomatosis with polyangiitis; a distinctive presentation with myocarditis and autoimmune haemolytic anaemia: case report

**DOI:** 10.3389/fcvm.2024.1490735

**Published:** 2025-01-14

**Authors:** Doaa Abdulwahab Mohammed Ayish, Fatma Ayish, Asayel Khamjan, Amal. H. Mohamed, Nagla Abdalghani, Osama Albasheer

**Affiliations:** ^1^ Medical and Rheumatology Consultant, Head of Medical Department in Jazan General Hospital, Jazan, Saudi Arabia; ^2^ Internal Medicine Department, Faculty of Medicine, Jazan University, Jazan, Saudi Arabia; ^3^ PGY4 Internal Medicine Residency Program, King Fahad Central Hospital-Jazan, Jazan, Saudi Arabia; ^4^ Respiratory Therapy Department, Faculty of Applied Medical Sciences, Jazan University, Jazan, Saudi Arabia; ^5^ Family and Community Medicine Department, Faculty of Medicine, Jazan University, Jazan, Saudi Arabia

**Keywords:** eosinophilia, myocarditis, vasculitis, autoimmune haemolytic anaemia, heart failure

## Abstract

**Background:**

Eosinophilic granulomatosis with polyangiitis (EGPA) is an extremely rare type of vasculitis characterized by inflammation within small blood vessels or tissues that may cause damage to the lungs, heart, kidneys, and other organs. Here, we present a rare case of EGPA with cardiac involvement that presented with acute heart failure.

**Clinical findings:**

A 44-year-old woman with a history of bronchial asthma and sinusitis presented with fever, shortness of breath, fatigue, unintentional weight loss, and polyarthritis. Physical examination revealed bilateral basal crepitation and mononeuritis multiplex.

**Diagnosis:**

The peripheral blood smear revealed red blood cells of different sizes and shapes (dimorphic features), more eosinophils, low hemoglobin, and higher lactate dehydrogenase (LDH) levels. Cardiac magnetic resonance imaging (CMR) revealed global hypokinesia and features suggestive of myocarditis. Echocardiography showed a low ejection fraction of 25%. Thus, the patient diagnosed with EPGA and myocarditis presented with acute heart failure.

**Interventions:**

The patient was administered high-dose corticosteroids (intravenous bolus methylprednisolone 500 mg for three days, followed by 1 mg/kg of prednisolone) and cyclophosphamide 750 mg intravenously.

**Outcome:**

After one months, the patient showed a marked improvement in clinical and laboratory parameters. The ejection fraction improved to 30%–40%, the eosinophil count returned to normal, and the haemolytic anaemia resolved. The patient was sent home and shifted to mycophenolate mofetil 1 g twice a day as maintenance therapy.

**Conclusion:**

Patients with EGPA have a higher morbidity and mortality rate when they have cardiac involvement. The pathophysiological mechanism of cardiac involvement in EGPA warrants consideration of immunosuppressive therapy in addition to standard heart failure treatment.

## Introduction

Eosinophilic granulomatosis with polyangiitis (EGPA), formerly known as Churg-Strauss syndrome (CSS), is a subtype of small-to medium-sized vessel anti-neutrophil cytoplasmic antibody (ANCA)-associated vasculitis. Necrotizing granulomatous inflammation, high concentration of eosinophils, and involvement of the respiratory tract are hallmarks of this condition (1).

The eosinophilic phase of the disease is characterized by eosinophilic organ infiltration in the heart, lungs, and digestive tract. This is the main sign of disease and is usually linked to ANCA-negative disease. The vasculitis phase is characterized by purpura, peripheral neuropathy, glomerulonephritis, and constitutional symptoms. ANCA-positive patients were more likely to have vasculitis features (2, 3).

Asthma usually precedes vasculitis symptoms (mean 9.3 + 10.8 years). The condition affects approximately 90%–100% of patients, and their features differ from those of other asthma patients. It is typically late-onset asthma, manifesting in adults between the ages of 30 and 40. Allergy-related upper respiratory tract symptoms, such as allergic rhinitis, chronic sinusitis (70%–90%), and nasal polyps, frequently coexist with asthma in patients with EGPA. The lungs are also affected by eosinophilic infiltration, and 70% of patients have peripheral reticulonodular or consolidative pulmonary opacities on chest x-rays. Pleural effusion and hilar or mediastinal lymphadenopathy are the two less common thoracic symptoms of EGPA (3).

Depending on the study (4), the percentage of heart involvement varies from 16.0% to 29.0%. Eosinophilia and its cytotoxicity are the main ways that EGPA hurts the heart. These patients have a higher eosinophil count at diagnosis, higher disease activity, negative ANCA, and higher C-reactive protein levels (3, 5).

EGPA is associated with an increased mortality risk and worsened prognosis in the presence of cardiac involvement. The primary cause of first-year mortality and overall mortality in EGPA is cardiomyopathy, which accounts for nearly one-third of the deaths (6). All patients should undergo periodic electrocardiography and echocardiography to detect any asymptomatic cardiac involvement early. Cardiac magnetic resonance (CMR) imaging is the gold standard method for diagnosing cardiomyopathies and assessing the staging of myocarditis (5).

In some rare cases, the symptoms of EGPA cardiomyopathy may be reversible with treatment, despite how bad they are. This shows how important it is to quickly find and treat heart problems (1, 5).

Warm-reacting IgM autoantibodies rarely cause autoimmune hemolytic anemia (AIHA). Warm IgM autoantibodies, while not directly linked to EGPA, have been associated with immune disorders like immune thrombocytopenic purpura, severe combined immunodeficiency, and Sjogren's syndrome. Warm IgM autoantibodies cause AIHA, which has a very poor prognosis and typically resists standard treatments for IgG-mediated hemolytic anemia. Generally, severe AIHA and significant anemia are associated with a highly positive direct antiglobulin test (DAT) (7, 8).

A significant proportion of patients with EGPA (42%–76%) has nervous system involvement and was ANCA-positive. Mononeuritis multiplex is the most common symptom, and it often affects the tibial, peroneal, median, and ulnar nerves (2, 3). Foot drop and symmetric polyneuropathy are common symptoms that worsen if the treatment is not initiated early. Up to 63% of patients describe pain, limb weakness, numbness, burning sensation, or other sensory disturbances as their initial symptoms. EGPA reports only 5%–29% of cases with neurological symptoms involving the central nervous system (CNS). 52% of cases had ischemic cerebrovascular lesions, caused by intracerebral and/or subarachnoid hemorrhage (24%), loss of visual acuity (33%), and cranial nerve palsies (21%) (3).

The diagnosis of EGPA is still difficult, in part because of the long-lasting nature of asthma and the need for long-term corticosteroids (CS), which can obscure other symptoms of the condition (3). Currently, the European Alliance of Associations for Rheumatology (EULAR) and the American College of Rheumatology (ACR) endorse the new EGPA classification criteria to validate diagnosis (Table 1) (1). This case report analyzed and described the clinical characteristics and outcomes of EGPA patients with myocarditis and AIHA, aiming to enhance the overall comprehensive understanding and provide useful information for clinical practice.

**Table 1:** The American college of rheumatology and the European alliance of associations for rheumatology (2022) classification criteria for EPGA (1).

2022 AMERICAN COLLEGE OF RHEUMATOLOGY/EUROPEAN ALLIANCE OF ASSOCIATIONS FOR RHEUMATOLOGY CLASSIFICATION CRITERIA FOR EOSINOPHILIC GRANULOMATOSIS WITH POLYANGIITIS	
CONSIDERATIONS WHEN APPLYING THESE CRITERIA	
• These classification criteria should be applied to classify a patient as having eosinophilic granulomatosis with polangitis when a diagnosis of small- or medium- vessel vasculitis has been made.	
• alternate diagnosis mimicking vasculitis should be excluded prior to applying the criteria	
CLINICAL CRITERIA	
Obstructive airway disease	+3
Nasal poyps	+3
Mononeuritis multiplex	+1
LABORATORY AND BIOPSY CRITERIA	
Blood eosinophilic count ≥1 × 109/L	+5
Extravascular eosinophilic- predominant inflammation on biopsy	+2
Positive test for cytoplasmin antineutrophil cytoplasmic antibodies (cANCA) or antiproteinase 3 (anti-PR3)	−3
Hematuria	−1
Sum the scores for 7 items, if present. A score of ≥6 is needed for classification of EOSINOPHILIC GRANULOMATOSIS WITH POLYANGIITIS	

## Case description

A 44-year-old Saudi female had history of bronchial asthma, allergic rhinitis, and sinusitis since she was 18 years old. She had experienced an attack of chest pain 27 days before presentation, for which she sought medical advice many times and received analgesia with partial response. Two days before admission, she presented to the emergency department as the chest pain became severe and more frequent and was associated with shortness of breath on mild to moderate exertion and bilateral lower limb swelling. She denied any other symptoms related to the cardiopulmonary system and had no history of cardiac disease. Her condition was accompanied by generalized fatigability, fever, and an unintentional weight loss of 3 kg. Furthermore, she described that the condition was associated with polyarthritis, as well as loss of sensation over the dorsum of the right foot and left thumb. However, she did not report any vasculitis or skin rash or gastrointestinal symptoms. Her regular medications included inhaled corticosteroids in addition to short-acting β agonist inhalers, as required.

**Figure 1:**
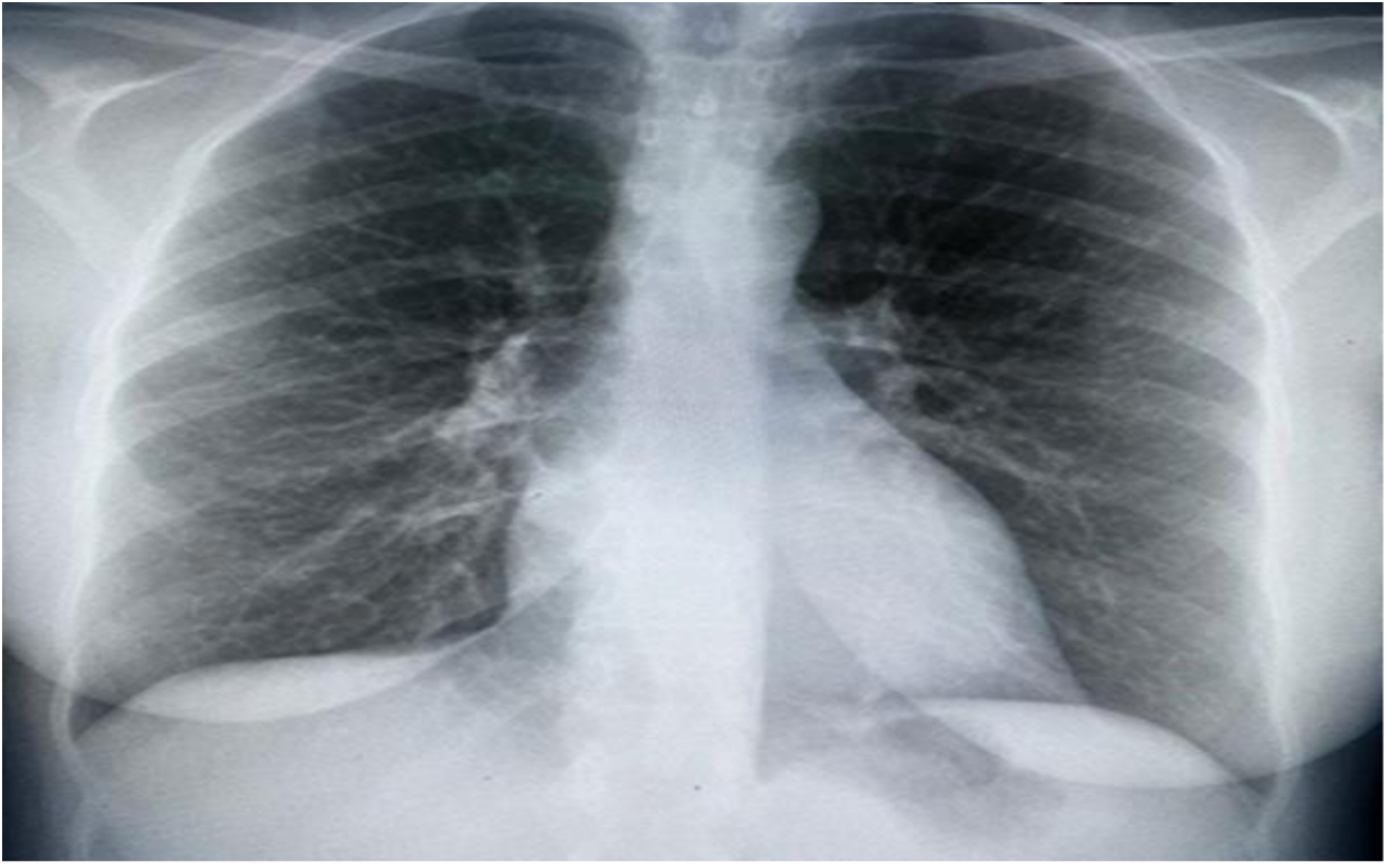
Chest x-ray showing bilateral interstitial opacities consistence with pulmonary congestion.

Upon assessment, the patient was in a semi-sitting position and slightly pale. Her pulse 110/min, blood pressure was 105/70 mmHg, and Spo2 was 95% on room air. Cardiovascular examination showed tachycardia, normal S1 and S2, no murmur, and no added sounds. Chest examination revealed symmetrical air entry with bilateral fine basal crepitation. Abdominal examination results were normal, with no organomegaly. Musculoskeletal examination was unremarkable, apart from bilateral lower limb edema. Neurological examination revealed sensory loss in the right dorsal foot and the left thumb. Electrocardiography (ECG) showed sinus tachycardia, no ischemic changes, or other abnormalities. Chest radiography revealed pulmonary congestion (Figure 1).

The patient was diagnosed with acute heart failure, admitted to a high dependency unit (HDU), and received oxygen and 80 mg intravenous (IV) furosemide as bolus dose followed by furosemide 40 mg intravenously for 12 h with 12 leads ECG monitoring and workup continued to look for the underlying cause with cardiology consultation.

During her three-day HDU hospitalization (Days 1–3), a thorough workup indicated low hemoglobin level (9.3 g/dl), leukocytosis with an elevated eosinophil count (4 × 10^9^/L; normal range: 0.2–0.5 × 10^9^/L), and evidence of neutrophilic predominance. The peripheral blood smear revealed severe, dimorphic hemolytic anemia, along with neutrophilic leukocytosis and monocytosis. The direct antiglobulin test (DAT) was positive on two separate occasions. Additionally, inflammatory markers were elevated, including an erythrocyte sedimentation rate of 109 mm/h and a C-reactive protein level of 96 mg/L. Cardiac enzymes and markers were also high: troponin was 2 μg/L (normal: 0.01–0.02 μg/L), lactate dehydrogenase (LDH) was 1,218 U/L, creatine kinase (CK) was 913 U/L, and creatine kinase–myocardial band (CK-MB) was 192 U/L. Renal, hepatic, and thyroid function tests were within normal limits, and virological screening as well as blood cultures were negative (Table 2).

**Table 2:** Laboratory work-up.

Investigations	Patient results	Normal range
WBC	17 × 10^9^/L	4.5–11.0 × 10^9^/L
Neutrophils	8 × 10^9^/L	2.5–6 × 10^9^/L
Lymphocytes	3.5 × 10^9^/L	1.0–4.8 × 10^9^/L
Eosinophil	4 × 10^9^/L	0.2–0.5 × 10^9^/L
Haemoglobin	9.3 g/dl	12–16 g/dl
Peripheral blood smear	Haemolytic anemia (dimorphic) of severe degree, Neutrophil leukocytosis and monocytosis.	
Creatinine	71 µmol/L	61.9–114.9 µmol/L
Hepatic profile	Normal	
ESR	109 mm/h	≤20 mm/h
CRP	96 mg/dl	<0.3 mg/dl
Hepatitis B surface antigen	Negative	
Hepatitis C antibody	Negative	
HIV	Negative	
LDH	1,218 units/L	105–233 units/L
CK	913 IU/L	30–145 U/L
CKMB	192 IU/L	5 and 25 IU/L
Tropnin	2 ug/L	(0.01–0.02) ug/L
TFT	Normal	
Blood cultures	Negative	
DAT	Positive in two occations	
ANCA	Negative	
Urine analysis	Negative for RBCs casts	

WBC, white blood count; ESR, erythrocyte sedimentation rate; CRP, C-reactive protein; HIV; human immune deficiency virus; LDH, lactate dehydrogenase; CK, creatine kinase; CKMB, creatine kinase myocardium bound; TFT, thyroid function test; DAT, direct antiglobulin test; ANCA, anti-neutrophil cytoplasmic antibody; RBC, red blood cells.

Transthoracic echocardiography (TTE) demonstrated significant left ventricular systolic dysfunction, reflected by a reduced ejection fraction of approximately 25%. In addition to global hypokinesia of the left ventricle, there were no significant valvular abnormalities or signs of pericardial effusion.

Given the extremely elevated eosinophil count, vasculitic pathology was thought to be the most likely diagnosis, and further investigations and rheumatologic work-up were initiated.

**Figure 2:**
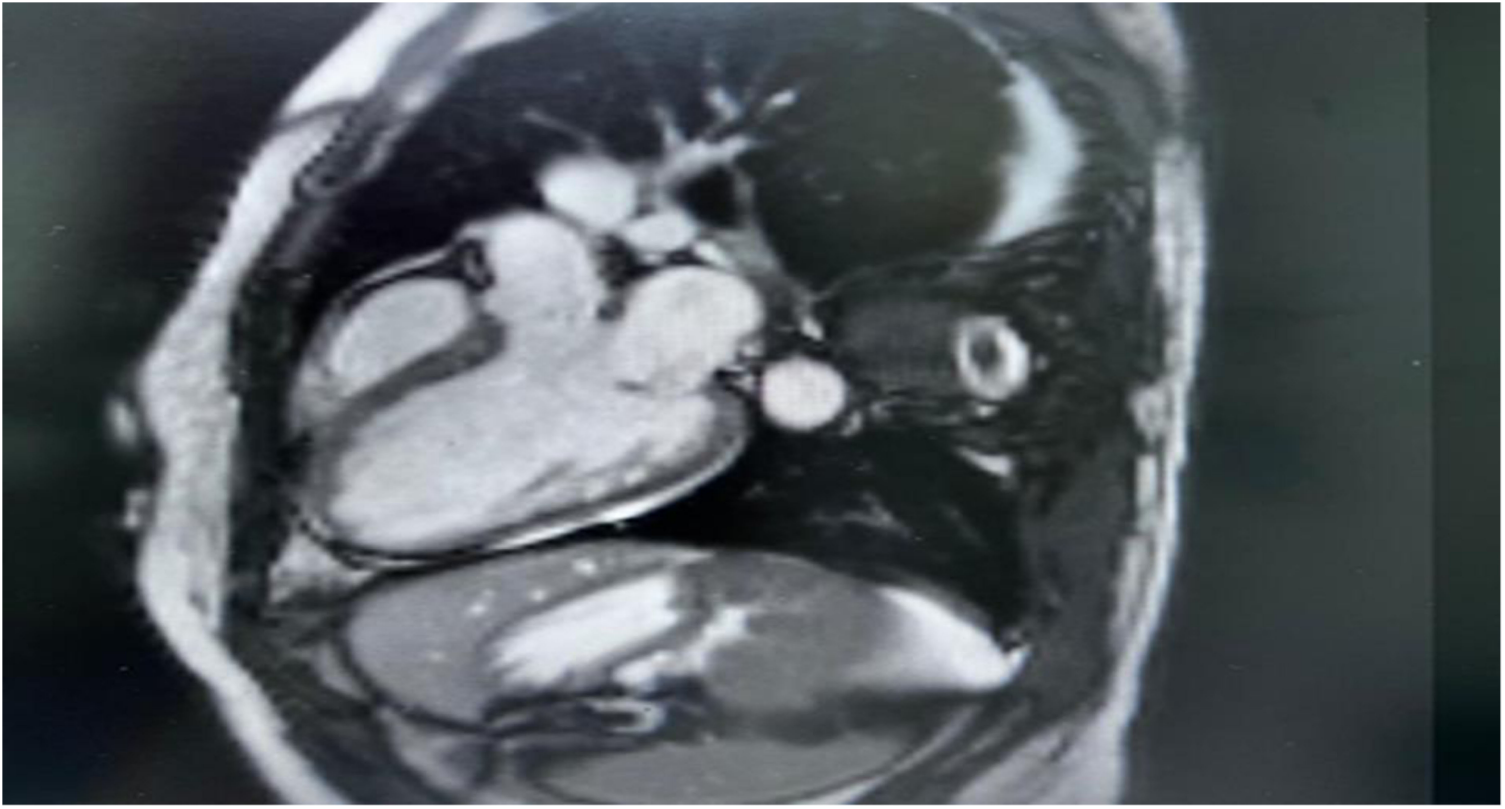
Cardiac MRI, there is non-territorial focal transmural delayed enhancement involving the anteroseptal wall of the mid myocardium. Also there is focal sub endocardial enhancement of the inferior-lateral wall of the mid myocardium. Small right-sided pleural effusion is noted.

Cardiac MRI imaging suggested myocarditis (Figure 2). Globally, hypokinemia occurs in the left ventricle with a mild to moderate reduction in systolic function. Focal delayed enhancement was observed along the anteroseptal wall of the mid-myocardium. The inferolateral wall of the mid-myocardium also showed focal sub-endocardial enhancement. Pericardial fluid or thickening was not observed. The right side showed a small pleural effusion. No mediastinal lymph nodes were observed.

**Figure 3:**
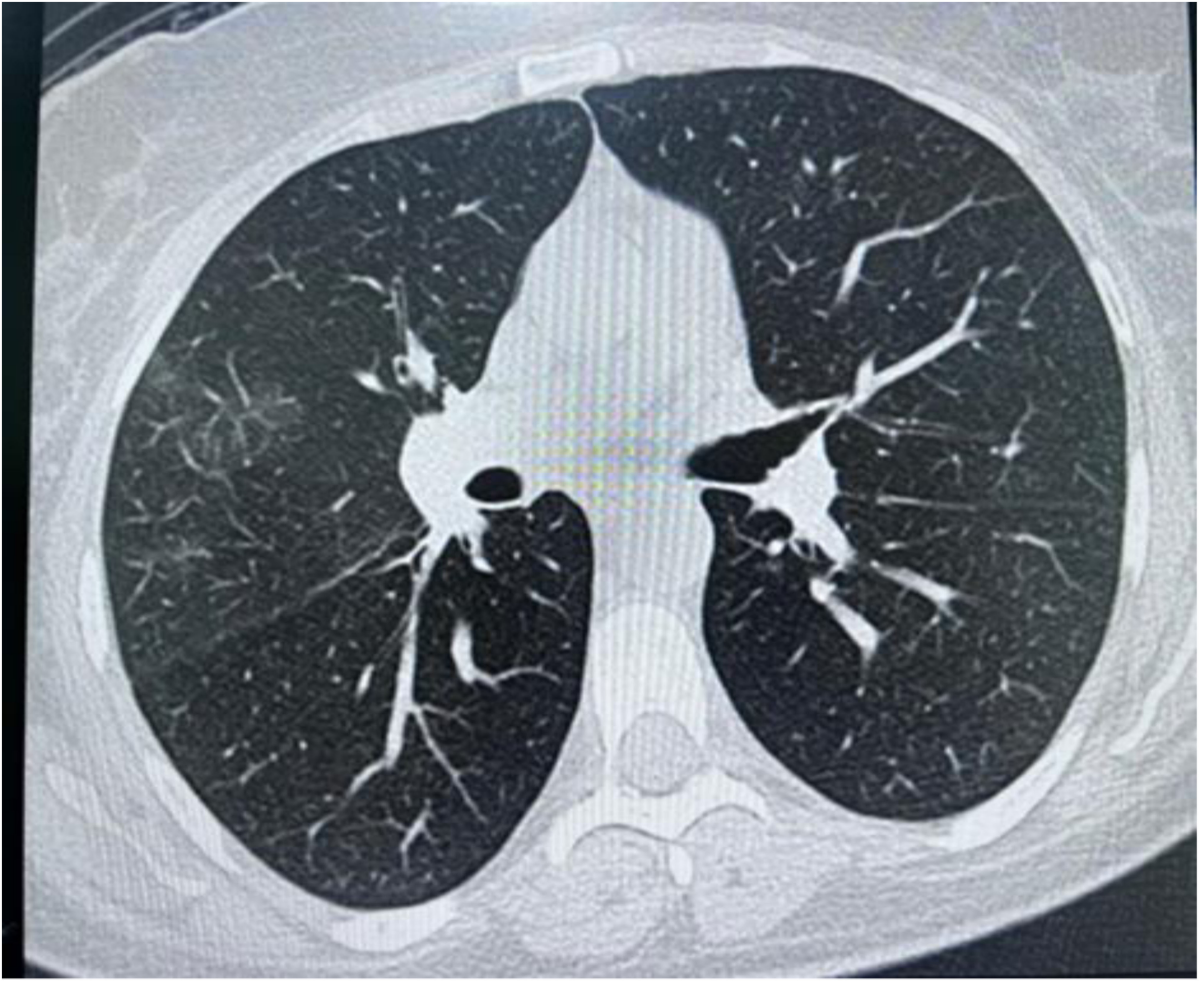
High resolution computed tomography (HRCT) chest showing peripheral and central faint patchy areas of ground glass densities within the lower segment of right upper lobe.

High-resolution computed tomography (HRCT) chest showed a few peripheral and central faint patchy areas of ground glass densities in the lower segment of the right upper lobe, with no signs of lung masses, consolidation, or cavitation (Figure 3). There was ground glass densities in the right upper lobe, possibly indicative of an inflammatory or infectious process. Furthermore, there was no mediastinal lymph node enlargement or pneumothorax.

With a background history of asthma, eosinophilia, chest involvement in HRCT, and the presence of neuropathy, diagnosis of EGPA was considered, and further investigations were conducted. ANCA was negative. Urine analysis was negative for blood and red RBCs casts (Table 2).

Brain CT positive for ethmoid sinusitis. While nerve conduction study showed normal latency and amplitude and slow velocity of 41 m/s for the right tibial nerve, and peroneal nerve showed normal latency and amplitude with slow velocity at 39 and 40 m/s, and both ulnar nerves showed normal latency amplitude and slow velocity at 38 and 46 m/s (Table 3). Thus, the patient was diagnosed with EGPA, myocarditis, autoimmune hemolytic anemia, and peripheral neuropathy.

**Table 3:** Diagnostic images and nerve conductive study.

Diagnostic modality	Result
Echocardiogram	Ejection Fraction 25%
CT brain	No acute territorial infarction. Preserved grey white matter differentiation. No intra cerebral focal lesion. No intracerebral hematoma or extra-axial collection. No shift of midline structures. Sulci, ventricles and basal cisterns are patent. Partially degraded images of posterior cranial fossa without suspicious lesion are seen. Orbits and mastoid air cells are unremarkable. No suspicious skull lesion. Mild ethmoid sinusitis is noted.
Nerve conduction study	Normal for median and ulnar nerve bilaterally. Right tibial nerve shows normal latency and amplitude and slow velocity at 41 m/s. No response could be obtained from right common peroneal nerve. Left tibial and left common peroneal nerve show normal latency, amplitude and slow velocity at 39 and 40 m/s. No sensory response could be obtained from both median nerve. Both ulnar nerve shows normal latency amplitude and slow velocity at 38 and 46 m/s. Findings suggestive of myelinic neuropathy both lower limbs and diffuse sensory neuropathy.

Treatment: The patient was administered high-dose corticosteroids (intravenous bolus methylprednisolone 500 mg for three days, followed by 1 mg/kg of prednisolone) and cyclophosphamide 750 mg intravenously according to the European Vasculitis Society (EUVAS) protocol (9). Before starting immunosuppressive treatment, the patient was screened for latent tuberculosis (TB) and Interferon-Gamma Release Assays (IGRA), which were positive; therefore, she was started on prophylactic rifampicin 600 mg for four months.

Following early stabilization in the HDU, the patient's health gradually improved. She was transferred to the general ward on Days 4–8, where her respiratory and cardiac state improved with supportive care and continuous immunosuppressive medication.

On Day 8, she was returned home in stable condition after significant clinical and laboratory improvements, including an ejection fraction increase to 30%–40%, normalization of eosinophil counts, and remission of hemolytic anemia. She was kept on oral prednisolone and planned for regular intravenous cyclophosphamide infusions, monthly for three months and periodic blood tests (Table 4).

**Table 4:** Overview of clinical presentation, diagnostic workup, and therapeutic interventions.

Timeframe	Events and clinical findings	Interventions/treatments	Outcomes/notes
18 years prior	Diagnosis of bronchial asthma, allergic rhinitis, and sinusitis	Inhaled corticosteroids, short-acting *β*_2_ agonists as needed	Long-term management of respiratory symptoms
27 days before admission	Intermittent chest pain	Analgesics as needed	Partial relief, no definitive diagnosis
2 days before admission	Severe, frequent chest pain, dyspnea on mild exertion, bilateral lower limb swelling, fever, fatigue, unintentional weight loss (3 kg), polyarthritis, and sensory changes (right dorsal foot & left thumb)	Presented to emergency department	Condition worsened, prompting further evaluation
On admission (Day 0)	Tachycardia, mild hypoxia, bilateral basal crepitations	Admitted to High Dependency Unit (HDU), started on oxygen therapy, IV furosemide (bolus + 12-h)	Initial stabilization and close monitoring
HDU stay (Days 1–3)	Investigations: low Hb, high eosinophils, positive DAT, elevated ESR/CRP, elevated cardiac enzymes, EF ∼25%. CMR: myocarditis. HRCT: patchy ground-glass opacities. Nerve conduction studies: peripheral neuropathy	High-dose IV methylprednisolone (500 mg ×3 days) then oral prednisolone (1 mg/kg), IV cyclophosphamide (750 mg), prophylactic rifampicin for latent TB	Diagnosis of EGPA with myocarditis and AIHA established; immunosuppression initiated
General ward (Days 4–8)	Clinical improvement, reduced inflammatory markers, improved cardiac status	Continued oral prednisolone (1 mg/kg), supportive care	Further recovery, preparing for discharge
Discharge (Day 8)	Marked improvement, EF increased to 30%–40%, normalization of eosinophil count, resolved hemolytic anemia	Discharged home on oral prednisolone (1 mg/kg) and scheduled IV cyclophosphamide infusions	Stable condition at discharge
3 months post-discharge	Continued improvement under outpatient follow-up	Transitioned to mycophenolate mofetil (1 g twice daily) for maintenance therapy	Sustained remission, reduced steroid dependence

During outpatient follow-up three months after discharge, she had persistent remission and additional clinical improvements. At this point, she was switched to mycophenolate mofetil (1 g twice daily) as a maintenance medication, with the goal of reducing long-term corticosteroid exposure while maintaining disease control. She is scheduled for a comprehensive one-year follow-up evaluation to monitor for potential relapse, assess long-term cardiac function, and ensure ongoing disease control.

### Patient perspective

Following her treatment and clinical improvement, the patient reported a marked alleviation of her symptoms. She expressed feeling significantly better, capable of resuming her usual daily activities, and noted an overall enhancement in her quality of life. During follow-up appointments, she presented in excellent spirits, free from the previously debilitating symptoms and demonstrating improved mood and functionality. This positive change underscores the importance of prompt diagnosis, comprehensive treatment, and supportive care in managing complex conditions like EGPA.

## Discussion

This case report describes the clinical presentation of EGPA with myocarditis, AIHA, and multiple mononeuropathies in a background history of asthma and sinusitis. EGPA is a frequent systemic vasculitis affecting the heart, which is usually accompanied by eosinophilia and negative ANCA (1).

The young age of this female patient rendered her more prone to developing cardiac-related EGPA disease. Several researches show that EGPA patients with cardiac involvement started the disease earlier than those without (with mean ± SD: 38.4 ± 10.5 vs. 42.1 ± 15.9 years, respectively (5, 10).

As our patient was female, this aligns with one study reporting that women were more likely than men to have cardiac involvement (11). While some studies found that cardiac involvement is more frequent in men (5, 12, 13). However, there was a greater number of female patients in the group with cardiac disease than in the group without cardiac involvement, suggesting that female patients in these studies had a greater incidence of cardiac disease.

The patient presented with atypical chest pain for three weeks, and received general nonspecific treatment until overt heart failure developed, which raised the suspicion of myocarditis as the underlying cause of her cardiac problem. Myocarditis, abrupt heart failure, as in this case, as well as coronary vasculitis, myocardial infarction, ventricular arrhythmias, and sudden cardiac death are frequently associated with EGPA (2, 5). Clinical heart failure was reported in a meta-analysis of 62 cases, with a common cardiac presentation in 51.6% and chest pain in 32.3% (12). Lopes et al. reported a patient who presented with fulminant eosinophilic myocarditis (13), while Sakurai et al. described a patient with severe EPGA associated with advanced atrioventricular block and cardiac arrest (14). A fraction of patients will either show subtle presentation or no symptoms (1). Dennert et al. (13) found that asymptomatic heart disease was more frequent than symptomatic heart disease but stated that all participants were in remission, in contrast to our patient who had an active disease. Consequently, in individuals with suspected EGPA, thorough examination of cardiac involvement is recommended.

Furthermore, patients with EGPA who experience cardiac involvement typically experience involvement in other organs, as it is evident from this case that patients with EGPA can present with cardiac, neurological, and hematological involvement (3, 5, 7, 15). Itagak et al. reported cardiac involvement alone, as the patient exclusively had cardiac involvement as the only organ damage linked with EGPA, with the exception of sinusitis and asthma, which represent the prodromal phase of the disease (16).

Cardiac disease was confirmed by clinical presentation, ECG, cardiac biochemical markers, and cardiac images in this patient, which is consistent with the literature and other studies (2, 5, 12, 17) Vasculitis rarely manifests itself as a major cardiac problem. Prior research has demonstrated that, although nerve involvement is common among individuals with EGPA, the heart and gastrointestinal systems are less commonly affected (10).

Cardiac involvement in patients with EGPA has a particularly extremely poor prognosis (13, 17). Comarmond et al. reported a mortality rate that was around four times higher than that of people without cardiac disease (18). When EGPA-induced myocardial injury is suspected, it is crucial to perform multimodality imaging, such as cardiac magnetic resonance (CMR), particularly when endomyocardial biopsy (EMB) is negative or unavailable (19). In this patient, the diagnosis was made based on CRM and prompt treatment was initiated earlier. This may have contributed to the patient's successful clinical outcome. Another factor that affected the prognosis in this case was the glucocorticoid treatment for bronchial asthma.

CMR imaging can help clarify the diagnosis and assess the degree of myocardial necrosis. However, EMB alone can provide conclusive diagnoses (3, 5).

Furthermore, the case demonstrated laboratory data consistent with autoimmune hemolytic anemia based on positive direct Coombs and DAT tests, with no previous history of hemolysis. AIHA is a rare and unusual presentation of EGPA, and very few cases have reported this association (7).

The patient responded well to glucocorticoid therapy plus cyclophosphamide and continued maintenance treatment with 1 g Mycophenolate mofetil twice a day. Patients with heart lesions and AIHA with EGPA require aggressive and prompt therapeutic approaches. This approach may enable the recovery of cardiac function and reduce the significant mortality associated with EGPA (20). However, in patients with heart failure or mononeuritis multiplex, this treatment alone is ineffective (15). Therefore, the patient continued maintenance treatment with 1 g of mycophenolate mofetil twice a day.

Early medical intervention can prevent potentially fatal episodes of heart disease and reduce the high mortality rate associated with EPGA-related myocarditis. Even in the absence of symptoms, a thorough cardiac evaluation should be carried out on an EPGA patient, as this will help avoid serious cardiac complications.

We report a case of myocarditis related to EPGA that responded well to standard corticosteroid-cyclophosphamide therapy; however, in some cases, this treatment is ineffective for EGPA patients who also have heart failure or mononeuritis multiplex (20). Therefore, the patient continued maintenance treatment with 1 g of mycophenolate mofetil twice a day.

Mycophenolate mofetil (MMF) is increasingly acknowledged as a suitable option for maintenance therapy in eosinophilic granulomatosis with polyangiitis (EGPA), especially as a steroid-sparing agent (4). High-dose corticosteroids effectively control active disease; however, their long-term use is linked to considerable adverse effects, such as metabolic complications, osteoporosis, hypertension, and an elevated risk of infections. Recent international guidelines and consensus documents on the management of EGPA highlight the necessity of minimizing cumulative corticosteroid exposure to mitigate toxicity.

The 2022 ACR/EULAR classification criteria and EUVAS treatment recommendations advocate for the use of non-glucocorticoid immunosuppressive agents to maintain long-term remission in ANCA-associated vasculitis (AAV), including EGPA (21, 22). Azathioprine and methotrexate have traditionally been utilized; however, mycophenolate mofetil has become increasingly accepted for its immunomodulatory effectiveness, advantageous side-effect profile, and role as a steroid-sparing agent.

In order to effectively manage EGPA, which may cause recurrent disease activity and delayed organ involvement, close and ongoing follow-up is crucial (2, 22). Regular monitoring of clinical symptoms, inflammatory markers, and imaging scans enables early diagnosis of recurrence via a scheduled one-year examination, as in this patient's case. Although this patient had a good response to the conventional combination of high-dose corticosteroids, cyclophosphamide induction, and mycophenolate mofetil maintenance, some EGPA patients may have disease resistant symptoms or unacceptable side effects (7). When patients do not reach remission or have repeated relapses, new targeted therapy and different immunosuppressant may be investigated.

Other ANCA-associated vasculitides have showed promise in patients who are cyclophosphamide refractory or cannot take long-term corticosteroids (2, 7, 22). One such possibility is rituximab (7). Biologic drugs that target the IL-5 pathway, such as mepolizumab, have also developed into effective steroid-sparing treatments for individuals with significant eosinophilic activity. With their potential for improved long-term safety profiles, these medicines may aid in the targeted treatment of eosinophilic inflammation. Reports have also shown that the anti-IgE monoclonal antibody omalizumab improves asthma control in EGPA and may help reduce the utilization of systemic steroids (2, 22). Rheumatologists, pulmonologists, cardiologists, and hematologists who are well-versed in the many presentations of rheumatoid arthritis should ideally work together as a multidisciplinary team to make personalized therapy recommendations for refractory EGPA.

## Conclusion

We report a case of myocarditis and AIHA related to EPGA that responded well to standard corticosteroid-cyclophosphamide therapy. As cardiac involvement substantially increases EGPA-related mortality and morbidity, early diagnosis and treatment can prevent patients from experiencing serious late-stage cardiac complications.

## Data Availability

The raw data supporting the conclusions of this article will be made available by the authors, without undue reservation.
